# Electrospun cellulose acetate/activated carbon composite modified by EDTA (rC/AC-EDTA) for efficient methylene blue dye removal

**DOI:** 10.1038/s41598-023-36994-5

**Published:** 2023-06-19

**Authors:** Nehad A. Elmaghraby, Ahmed M. Omer, El-Refaie Kenawy, Mohamed Gaber, Mohamed A. Hassaan, Safaa Ragab, Ismail Hossain, Ahmed El Nemr

**Affiliations:** 1 Environment Division, National Institute of Oceanography and Fisheries (NIOF), Kayet Bey, Elanfoushy, Alexandria Egypt; 2 Polymer Materials Research Department, Advanced Technology and New Materials Research Institute (ATNMRI), City of Scientific Research and Technological Applications (SRTA-City), P.O. Box 21934, New Borg El-Arab City, Alexandria Egypt; 3 Department of Chemistry, Faculty of Science, University of Tanta, Tanta, 31527 Egypt; 4 School of Natural Sciences and Mathematics, Ural Federal University, Yekaterinburg, Russia 620000

**Keywords:** Environmental chemistry, Organic chemistry, Polymer chemistry, Environmental chemistry

## Abstract

The present study fabricated regenerated cellulose nanofiber incorporated with activated carbon and functionalized rC/AC3.7 with EDTA reagent for methylene blue (MB) dye removal. The rC/AC3.7 was fabricated by electrospinning cellulose acetate (CA) with activated carbon (AC) solution followed by deacetylation. FT-IR spectroscopy was applied to prove the chemical structures. In contrast, BET, SEM, TGA and DSC analyses were applied to study the fiber diameter and structure morphology, the thermal properties and the surface properties of rC/AC3.7-EDTA. The CA was successfully deacetylated to give regenerated cellulose nanofiber/activated carbon, and then ethylenediaminetetraacetic acid dianhydride was used to functionalize the fabricated nanofiber composite. The rC/AC3.7-EDTA, rC/AC5.5-EDTA and rC/AC6.7-EDTA were tested for adsorption of MB dye with maximum removal percentages reaching 97.48, 90.44 and 94.17%, respectively. The best circumstances for batch absorption experiments of MB dye on rC/AC3.7-EDTA were pH 7, an adsorbent dose of 2 g/L, and a starting MB dye concentration of 20 mg/L for 180 min of contact time, with a maximum removal percentage of 99.14%. The best-fit isotherm models are Temkin and Hasely. The outcome of isotherm models illustrates the applicability of the Langmuir isotherm model (LIM). The maximal monolayer capacity *Q*_m_ determined from the linear LIM is 60.61 for 0.5 g/L of rC/AC3.7-EDTA. However, based on the results from error function studies, the generalized isotherm model has the lowest accuracy. The data obtained by the kinetic models’ studies exposed that the absorption system follows the pseudo-second-order kinetic model (PSOM) throughout the absorption period.

## Introduction

Many pollutants, including dyes and other chemicals, are disposed of in water bodies that cause severe environmental contamination and harm to human and other living organisms' health due to their high toxicity^1–3^. Dyes are used in several industrial processes involved in textile, photography, paper-printing, cosmetics, leather-dyeing, food, rubber, plastics and the pharmaceutical industry for coloring their products^4–6^. About 10 to 15% of the produced dyes are released as effluent during dyeing^7,8^. Methylene blue (MB) dye is a cationic dye with a chemical formula of C_16_H_18_N_3_SCl, and it was used as a chemical analysis indicator, medicine to cyanide poisoning, biological aspects, and aquaculture^9,10^. MB dye effluent can severely damage fertile human health, fisheries and agricultural land^11^. Diverse methods are used to remove dyes, including chemical remediation, adsorption, flocculation, biological treatment, electrochemical, precipitation, coagulation, advanced oxidation and photocatalysis^8,12–14^. The most efficient method to uptake dyes from water is biological degradation, chemical remediation and adsorption^15^. However, some of these methods have limits, such as less bio-efficiency for biological degradation and chemical remediation methods are costly and unsuitable for complex dye^16^. Adsorption techniques are reported as the most efficient, easy to handle, reversibility, low-cost, convenient recycling and safe method for removal of undesired toxic dyes^8,17^. Cellulose is the most naturally abundant polysaccharide bio-materials consisting of hundreds to thousands of *β*-linked D-glucose units^18–20^. It has appealing properties such as being renewable, environmentally friendly, cost-effective, non-toxic, bio-degradable, bio-compatible, having a great degree of crystallinity, an extraordinary degree of polymerization, an elevation tensile strength, being highly crystalline, and having a high specific surface area^21–23^. Notable investigations on cellulose acetate & nano-clay composites on electrospinning were undertaken; however, an effective approach to properly using cellulose acetate and clay for removing pollutants is still lacking^24–26^.

In recent studies, cellulose nanomaterials have gained wide attention to be used as natural bio-based adsorbent materials because of their possible regeneration, great uptake capacity, great surface area, and accessibility of these materials in great quantity worldwide^27^. Moreover, electrospinning is a simple, eco-friendly, and low-cost technology for producing nanofibers with a high porosity ratio^28^. Since cellulose is poorly soluble in conventional solvent systems, fabricating cellulose nanofibers using electrospinning has proven difficult^29^. The formation of cellulose acetate (CA) nanofibers followed by a deacetylation process is considered the most suitable method to obtain cellulose nanofibers (CNFs). Cellulose nanofiber possesses a high porosity and chemical stability that enable it to be used in different uses such as drug delivery, tissue engineering, electrodes, wastewater purification and food packaging^30^. Adding inorganic agents, including graphene oxide, carbon nanotube and activated carbon, to cellulose nanofiber can improve its low mechanical resistance and possess a high surface area^31^. Activated carbon (AC) is a porous material with a high adsorption capacity and a large specific surface area. While AC possesses active pores that allow for the well-organized removal of organic dyes and contaminants, its discharge into the environment may cause problems^32,33^.

Additionally, surface modification techniques that incorporate the necessary functional groups for organic dyes or heavy metal ions might improve the absorption effectiveness of cellulose nanofiber. A well-known chelating agent called ethylenediaminetetraacetic acid (EDTA) adsorbed on bio-polymers functions as a scavenge-chelating agent that can capture cationic dyes through many potent linkages^34^.

In this study, we fabricated rC/AC 3.7-EDTA nanofiber composite by electrospinning of CA/AC3.7 followed by deacetylation to obtain rC/AC3.7 and then modified with EDTA anhydrous to give rC/AC 3.7-EDTA. The obtained rC/AC 3.7-EDTA adsorbent was investigated for the absorption of MB dye from water. To the author's knowledge, the fabricated rC/AC3.7- EDTA composite is used for the first time to remove MB dye.

## Materials and methods

### Materials

ALPHA Chemie of India provided the CA (acetyl content: 29–46%, Mn: 50,000). *n*-Hexane (95% pure) was obtained from M-TEDIA. Activated Carbon (AC) was obtained from Fisher. Polyethylene glycol (PEG) was obtained from ACROS Organic (Mw = 200). Central Drug House provided acetone of HPLC grade (assay 99.8%). Merck provided *N,N-*dimethylacetamide (DMAA). CH_3_CH_2_OH was obtained from Inter. Co. for sup & MED, Industries, Egypt. Disodium salt dehydrate of Ethylenediaminetetraacetic acid was obtained from Sigma-Aldrich. Pyridine was obtained from Loba Chemie, and NaOH (Min. Assy 96%) was obtained from ADWIC, El Nasr Pharm. Chem. Co., Egypt). HCl (Assay 30–34%) was obtained from SD Fine-Chem Limited (SD FCL), Mumbai, India, and MB dye (basic blue 9; C.I.52015, C_16_H_18_N_3_ClS.xH_2_O, Mw = 319.85 g) was obtained from Honeywell Riedel-de Haën AG, SEELZE-HANNOVER, Germany. CH_2_Cl_2_ (HPLC grade) was obtained from Fisher chemicals, and all the reagents were used as received without further purification.

### Formation of CA/AC3.7 nanofiber composite

The CA/AC3.7 nanofiber composite was prepared by an electrospinning process, as described by Elmaghraby et al.^35–37^.

#### Preparation of cellulose acetate/activated carbon solution

CA/AC composite was obtained by dissolving CA in DMA/acetone (4:1) (v/v) mixture by continuous stirring for 2 h at room temperature (24 ± 2 °C) followed by ultra-sonication overnight at room temperature to obtain a homogenous solution. The ratio of solid materials to the solvent mixture was 10% (w/v). PEG facilitated the electrospinning process and overcame the solution mixture surface tension. The PEG to CA ratio was 1:1 wt/wt. AC with 3.7 percentage of the total cellulose acetate weight were added to CA solution^35–37^.

#### Electrospinning of CA/AC solutions

A + 26 kV voltage was used at the injector, and –10 kV voltage was applied for the collector. A polymer solution intended for electrospinning was inserted into a syringe with a volume of 20 mL and a needle made of metal with a blunt end. At a temperature of 24 − 2 °C, the syringe pump was programmed to deliver the polymer solution at a rate of 10 mL/min with a tip-to-collector distance of 10 cm. The webs were collected on a square collector made of aluminum. After removing the nanofiber mats from the collector and washing them with ethanol and water to completely remove the PEG, the mats were dried in a vacuum oven at 50 °C for 24 h^35–37^.

### Functionalization of cellulose/AC nanofiber composite

It involves the deacetylation of CA/AC composite to yield regeneration CA/AC composite, then chemically modification of r-C/AC composite with ethylenediaminetetraacetic acid (EDTA).

#### Deacetylation of CA/AC composite

By adding 0.1 M NaOH aqueous solutions and ethanol (EtOH) to the composite in an ultrasonic bath for 2 h, hydrolysis of the CA/AC composite nanofiber mats was accomplished. Acetyl groups' hydrolysis was stopped by rinsing with DW until the pH reached neutral. The deacetylated membranes were dried at 50 °C for 24 h.

#### Synthesis of EDTA dianhydride

**Figure 1:**
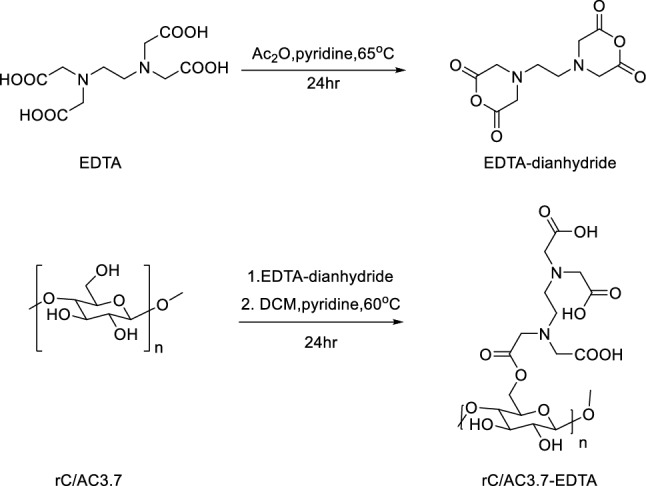
Scheme of preparation of the r-C/AC-EDTA nanofiber composite.

Ac_2_O (40 mL) was added to 30.0 g EDTA in 50 mL pyridine under N_2_. After being agitated for 24 h at 65 °C, the mixture was produced, cooled to room temperature, and filtered under a stream of N_2_ gas. The solid recovered was washed with 250 mL of anhydrous ethyl ether and dried overnight at 50°C (Fig. 1)^38^.

#### Preparation of r-C/AC-EDTA

About (1.0 g) r-C/AC nanofiber composite mats were dipped in 100 mL of dichloromethane containing EDTA dianhydride (3.0 g) and pyridine (5 mL). The mixture was shaken on shaker incubation for 20 h at 50 °C. Before being dried in an oven, the mats were washed with distilled water and ethanol (Fig. 1)^38^.

### Phisco-chemical characterization of the electrospun polymer nanofibrous composite

Surface morphologies of the composite were investigated using scanning electron microscopy (SEM) (JEOL, Model JSM 6360LA, Japan). Samples were coated using a thin gold layer before SEM observation. The electrospun fibers' average fiber diameter (AFD) was obtained using an Image-G Program. Surface compositions of the nanofibers were verified by Fourier transform infrared spectra (FTIR), which were carried out by using Bruker VERTEX70 connected to platinum ATR unit (model V-100) in the wavenumber range from 400 to 4000 cm^−1^. A thermal Analyzer is used to analyze the fabricated nanofiber. The sample was heated at 10 °C/min from room temperature to 900 °C under 100 mL/min N_2_ flow. Differential scanning calorimetric analyses (DSC) were carried out using SDT 650 Simultaneous Thermal Analyzer.

### MB dye adsorption

MB dye (1.0 g) was dissolved in 1000 mL of DW to make a stock solution containing 1000 mg/L of the dye. Using the batch equilibrium approach, 100 mL of the absorbate solution was shaken in a shaker while various dosages of the produced adsorbent were added. 0.5 mL of the supernatant solution was withdrawn after a specified time; the supernatant was tested for the leftover MB dye concentration by UV–visible spectrophotometer at the absorption wavelength of 665 nm. The absorption capacities of adsorbents were calculated using Eq. (1)

1 $$q_{{\text{t}}} = \frac{{C_{0} - C_{t} }}{m} \times V$$

where *q*_t_ is the absorption capacity of the r-C/AC-EDTA at time *t* (mg/g); *C*_0_ (mg/L) is the starting dye concentration, *C*_t_ is the remaining dye concentration after absorption has taken place over a time *t* (min.), *V* (L) is the dye solution volume in the shaking flask, and *m* (*g*) is the mass of the r-C/AC-EDTA. Equation 2 determines the percentage of dye removed from the water.

2 $${\text{Removal}}\;\left( \% \right) = \frac{{C_{0} - C_{t} }}{{C_{0} }} \times 100$$

### Experimental variables

#### Impact of pH

The impact of starting pH on MB dye absorption was investigated by adding 0.1 g of rC/AC3.7-EDTA to 100 mL of 60 mg/L MB dye solutions with a starting pH range (1–10). To change pH levels, 0.1 M HCl and NaOH solutions were utilized. The mixtures were shaken (200 rpm) for 210 min at room temp. and samples were taken for MB dye color analysis^39^.

#### Impact of starting MB dye concentration

For the rC/AC3.7-EDTA isotherm investigation, different MB solution concentrations (10–100 mg/L) were used. 0.05 to 0.25 g of the rC/AC3.7-EDTA were combined with 100 mL of adsorbate solutions with diverse MB dye concentrations, and the mixture was shaken (200 rpm) for 210 min at room temp.

#### Impact of absorbent dosage

The impact of rC/AC3.7-EDTA dose on MB dye removal was investigated by shaking 100 mL of (10–100 mg/L) for rC/AC3.7-EDTA of MB dye solutions with diverse doses of absorbents (0.05, 0.1, 0.15, 0.20, 0.25 g) for 210 min at room temperature.

#### Effect of contact time

Different weight from rC/AC3.7-EDTA (0.05, 0.10, 0.15, 0.2 and 0.25 g) was added to 300 mL glass flasks containing 100 mL of 10 to 100 mg/L MB dye solutions. Fast filtration was used to remove samples from the suspensions at various time intervals for 240 min while the suspensions were shaking at 200 rpm at room temperature^40^.

#### Effect of temperature

The experiments involved adding 0.1 g of rC/AC3.7-EDTA to 100 mL of water solutions of MB dye with 60 mg/L starting concentration and running them for 210 min at the temp of 298 K, 303 K, and 313 K. The following Eqs. (3)–(5) were used to further determine the enthalpy change (*H*^0^), Gibbs free energy change (*G*^0^), and entropy change (*S*) as the thermodynamic parameters

3 $$\Delta G^{0} = {-}RTln\left( {\frac{{Q_{e} }}{{C_{e} }}} \right)$$

4 $$\Delta G^{0} = \Delta H^{0} - T\Delta S^{0}$$

5 $$\ln \left( {\frac{{Q_{e} }}{{C_{e} }}} \right) = \frac{{\Delta S^{0} }}{R} - \frac{{\Delta H^{0} }}{RT}$$

where *T* (K) is the absolute temperature, *Q*_e_/*C*_e_ is the equilibrium distribution coefficient, and *R* is the universal gas constant (8.314 J/mol K). The Δ*G*^0^ values were obtained by calculating from Eq. (5). Values of *H*^0^ and *S*^0^ can be calculated from the Van’t-Hoff plot of ln(*Q*_e_/*C*_e_) versus 1/*T*.

### Absorption isotherm studies

The absorption isotherm explains the interaction of solutes with absorbents and optimises the absorbent application. As well as it describes the circulation mechanism of the absorption molecules between the liquid phase and the solid phase at the absorption equilibrium state.

### Best-fit isotherm model

A wide range of possible error functions has been investigated to investigate which isotherm model best matches the experimental equilibrium data. The error functions are applied: symbol N represents the quantity of experimental data points, and P represents the quantity of isotherm model parameters^41^.

### Kinetics studies

The rate of methylene blue absorption is explained by sorption kinetics, and this rate regulates the sorption equilibrium time. Dye sorption kinetics were necessary to choose the best operating parameters for the full-scale batch operation. The kinetic parameters that predict the sorption rate are useful for process design and modelling. As a result, pseudo-first-order equations were used to examine the kinetics of methylene blue dye sorption on the rC/AC3.7-EDTA nanofiber mat^42^, pseudo-second-order^43^, Elovich^41^ kinetic models. The experimental data and the values predicted by the model were correlated using correlation coefficients (*R*^2^, values near or equal to 1); the model with a comparatively higher value is better appropriate to the kinetics of MB dye sorption.

## Result and discussion

### Preparation of the rC/AC nanofiber composite-grafted EDTA

The deacetylation of cellulose acetate nanofiber to give regenerated cellulose make the hydroxyl group into a cellulose chain more readily react. Sven gram of CA/AC composite gives 3.7 g of rC/AC, meaning cellulose was completely deacetylated. FTIR analysis discussed below as acetyl peak around 1700 cm^−1^ wholly disappeared. On the other hand, dense hydrogen bonds within the cellulose chains make the –OH of glucose units less reactive^38^. In order to give cellulose nanofiber a decent level of functionalization, more reactive acid chlorides or anhydrides are utilised. We studied the functionalization of the regenerated cellulose nanofiber/activated carbon composite with the highly reactive EDTA dianhydride^44^. The rC/AC-EDTA was thoroughly washed in EtOH, DW, acetone, and then CH_2_Cl_2_ to eliminate all traces of the adsorbed substances. The esterification yield was 86.17% means that for every ten glucose units, around eight alcohol functionalities were esterified with EDTA molecules^38^.

### Phisco-chemical characterization of cellulose/activated carbon nanofiber composite

#### SEM analysis

**Figure 2:**
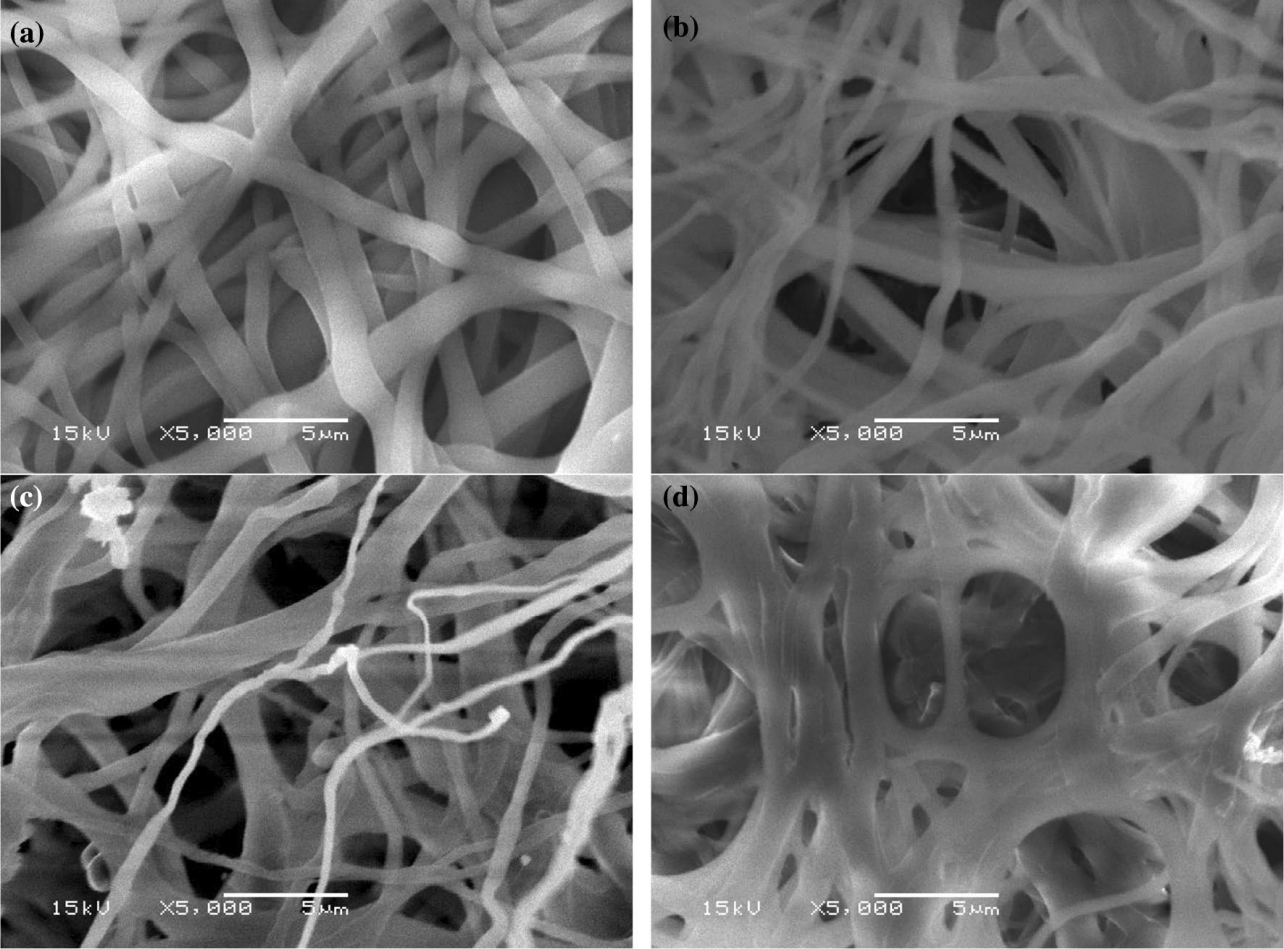
Images of SEM for (**a**) CA/AC3.7, (**b**) rC/AC3.7, (**c**) rC/AC3.7-EDTA and (**d**) rC/AC3.7-EDTA-MB; the images were taken at 15 kV and magnification of 5000 × .

The fiber diameter distribution and morphologies of CA/AC3.7, rC/AC3.7, rC/AC3.7-EDTA and rC/AC3.7-EDTA-MB dye nanofiber mats were displayed in Fig. 2. The morphology structures were bead-free cylindrical and smooth fibers, with submicron diameters are obtained. AFD for CA/AC3.7, rC/AC3.7, rC/AC3.7-EDTA and rC/AC3.7-EDTA-MB was 740, 564, 572 and 1118 nm, respectively. After the deacetylation process, the mat retained its shape, and the fiber diameter was dramatically shrunk as the acetyl group was removed. In contrast, the fiber diameter increased with the adsorption of methylene blue dye to 1118 nm^45^.

#### FT-IR spectra

**Figure 3:**
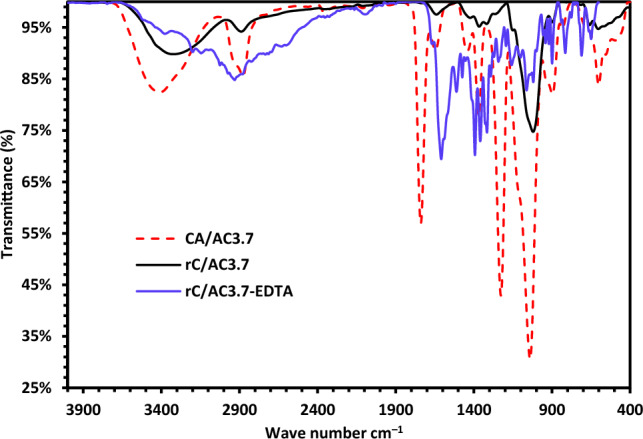
FTIR spectra of CA/AC3.7, rC/AC3.7 and rC/AC3.7-EDTA.

Figure 3 shows the FTIR spectra of CA/AC3.7, rC/3.7AC, rC/3.7AC-EDTA and AC samples. The wide band of about 3394 cm^−1^ is attributed to O–H stretching vibration, and the CA/AC3.7 composite sample showed the same characteristics. The stretching vibration of the C = O in the ester causes the absorption peak at 1737 cm^−1^ to exist^46^. The 1228 and 1368 cm^−1^ peaks are recognized as C-O and C-H bending vibrations, respectively. A strong peak at 1050 cm^−1^ was allocated to the C-O stretching in CA. The FTIR spectra registered of activated carbon displayed three bands around 3407, 1200 and 1500 cm^−1^, which are allocated in turn to (O–H) vibrations, (C–C) vibrations, and (C–O) stretching vibrations^47^. It was obviously seen the disappearance of the characteristic peaks of CA/AC3.7 that are recognized for the vibrations of the C=O of the acetate group at 1737 cm^−1^, C–CH_3_ at 1368 cm^−1^ and C–O–C at 1228 cm^−1^ in the rC/AC3.7^47^.

Both the spectra of rC/AC3.7 and rC/AC3.7-EDTA showed characteristic cellulose backbone absorption bands. For instance, the chemically modified cellulose nanofiber exposes a new peak at 1608 cm^−1^ that is evidence for forming a newly ester linkage between cellulose and EDTA, indicating the deformation of carbonyl (CO) groups of the carboxylic acid. Moreover, the peak at 1091 cm^−1^ was attributed to the C–O ester group vibrations, another proof of a successful functionalization reaction. The two absorption peaks at 1511 and 1454 cm^-1^ represent the asymmetric and symmetric OCO stretching modes of the carboxylate groups, respectively, and provide additional evidence that EDTA was successfully incorporated into the cellulose backbone^47–50^.

#### Thermal stability

**Figure 4:**
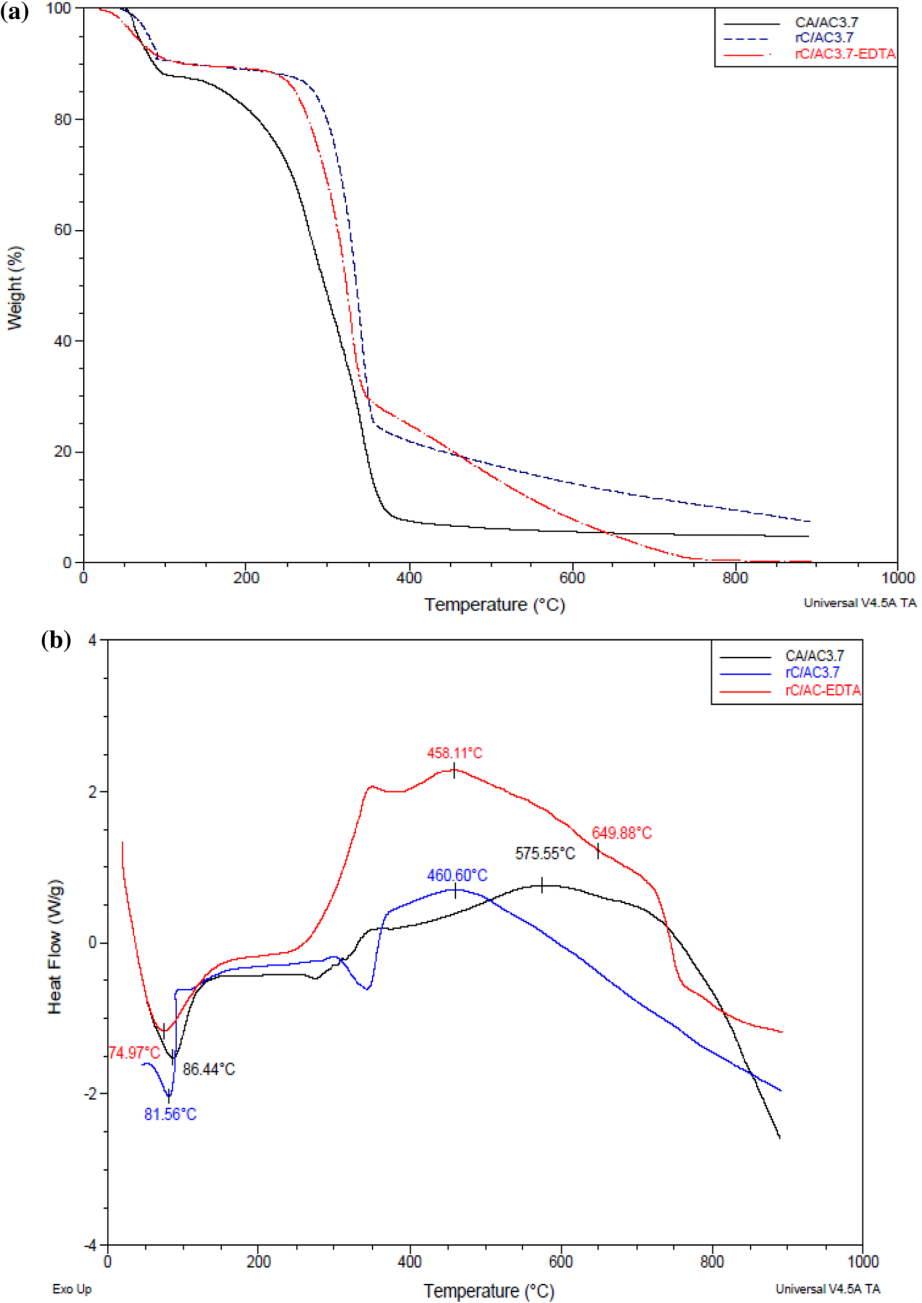
(**a**) TGA curvatures and (**b**) DSC curvatures of the CA/AC3.7, rC/AC3.7 and rC/AC3.7-EDTA composites nanofibers at temp range (50–900 °C) under 100 mL/min of N_2_ flow.

The impact of EDTA on the therml stability of rC/AC3.7 was evaluated by thermogravimetric analysis shown in Fig. 4a. The TGA behavior of CA/AC3.7 and rC/AC3.7 involves two thermal transitions, while rC/AC3.7-EDTA showed three thermal steps. The first thermal step represents the loss of moisture at low temp. (i.e., 80–120 °C) with mass losses 12.42, 9.238 and 9.054% for CA/AC3.7, rC/AC3.7 and rC/AC3.7-EDTA, respectively. Where the second thermal step represents the thermal degradation of cellulose or cellulose acetate at high temperatures (i.e., 120–460 °C) with weight losses of 79.57, 70.81 and 56.15% for CA/AC3.7, rC/AC3.7 and rC/AC3.7-EDTA. One more thermal step for rC/AC3.7-EDTA represented the thermal degradation of EDTA at a temperature of (480–700 °C) with a weight loss of 31.04%^51^. On the other hand, DSC analysis of CA, CA/AC3.7, rC/AC3.7 and rC/AC3.7-EDTA were shown in Fig. 4b. All samples displayed a crystallization endo peak representing a crystallization temperature at 86.44, 81.56 and 74.97 °C for CA/AC3.7, rC/AC3.7 and rC/AC3.7-EDTA, respectively, which are ascribed to water loss from the sample. The exo-melting band characterized a degradation temp of CA/AC3.7, rC/AC3.7, and rC/AC3.7-EDTA were 48.11, 575.55 and 460.60 °C, respectively, that was ascribed to the degradation of CA. rC/AC3.7-EDTA exhibited one additional peak at 649.88 °C attributed to the degradation of EDTA. The result showed that functionalization of regenerated cellulose with EDTA caused a slight decrease in the decomposition temperature of the nanofiber from 460.6 to 458.11 °C for the rC/AC3.7 and rC/AC3.7-EDTA samples, respectively. Including the C-N bond degradation at a lower temp in cellulose may cause a decrease in cellulose’s thermal stability during its modification by EDTA. Also, rC/AC3.7-EDTA exhibited one more peak corresponding to the thermal degradation of EDTA at 649.88 °C^52^.

### Batch absorption investigation

#### Impact of pH

**Figure 5:**
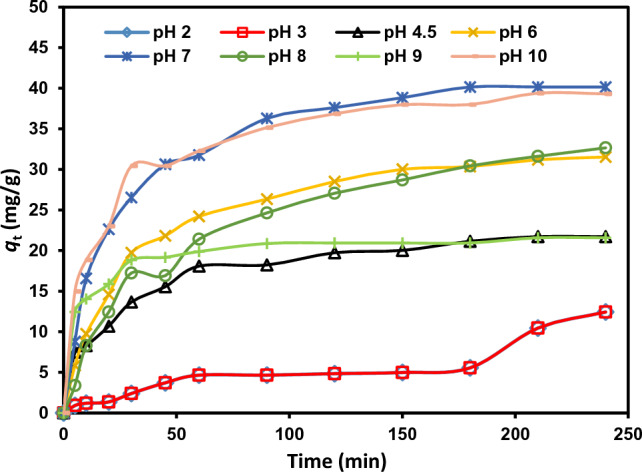
Impact of pH on absorption of MB dye (60 mg/L) onto rC/AC3.7-EDTA (1.0 g/L) at 25 ± 2 °C.

The dye adsorption efficiency depends on the solution pH level because the number of protons impacts the absorbent surface charge and the dye's ionization level. The absorption capacities of the rC/AC3.7-EDTA were studied at different pH of MB dye solution. Solutions containing 60 ppm of MB dye were shaken with the rC/AC3.7-EDTA (1 g/L) at room temp for (5 to 210) min, and the final dye concentration was measured by UV spectroscopy. The cationic dye, known as MB dye, produced positively charged ions when dissolved in water. The amount of absorption onto the adsorbent surface is primarily determined by the surface charge of the absorbent, which is controlled by the pH of the solution. Figure 5 illustrates the impact of pH on the percentage of MB dye absorption, showing a dramatic increase in absorption in the pH range of 7 to 10. At low pH, the protons concentration increase, so it competes with dye cations for binding in the active sites of the modified nanofiber; as a result, the adsorption capabilities of the modified nanofiber rapidly decline. Additionally, the surface of cellulose becomes positively charged at high proton concentrations, making dye cations less likely to approach due to electrostatic repulsions. As a result, at low pH, the absorption is relatively poor.

In contrast, at high pH, the carboxyl groups of rC/AC3.7-EDTA may deprotonate, generating negatively charged adsorption sites, and the cellulose surface may become negatively charged due to OH adsorption. At high pH levels, electrostatic forces of attraction strongly encourage adsorption. This study found the maximum absorption at pH 7 and 10 with high *q*_t_ 40.15 mg/g for pH 7. To imitate actual environmental situations, where the water pH is normally in the range of 6.5 to 7.5, our further experiments were carried out at pH 7, comparable with the literature results^41,53–55^.

#### Impact of adsorbent dosage

**Figure 6:**
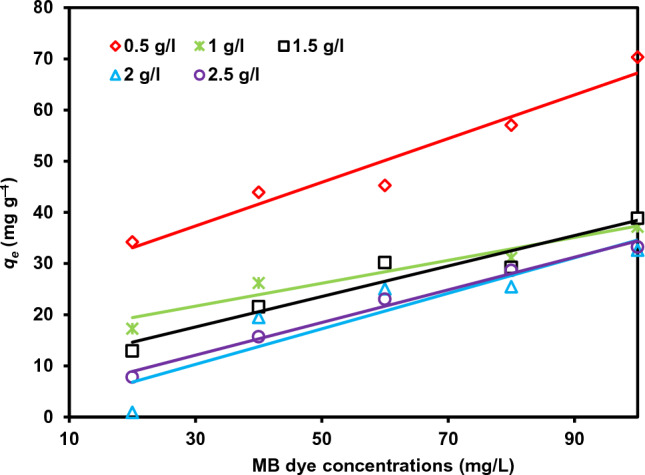
Impact of rC/AC3.7-EDTA dosage (0.5–2.5 g/L) on MB dye (*C*_0_: 20, 40, 60, 80 and 100 mg/L) absorption using rC/AC3.7-EDTA (0.5, 1.0, 1.5, 2 and 2.5 g/L, pH = 7, *t*: 210 min, T: 25 °C).

By adjusting the dosage from 0.1 to 0.25 g, it was possible to examine the impact of rC/AC3.7-EDTA adsorbent dose affected the absorption of different MB dye concentrations, as displayed in Fig. 6. For MB dye concentration for 20–100 mg/L with increasing the amount of rC/AC3.7-EDTA dosage from 0.05 to 0.25 g, the removal % increased from 85 to 99%, 45 to 97%, 37 to 95%, 35 to 89% and 35 to 83%, respectively. Therefore, regardless of the initial MB dye concentration, for rC/AC3.7-EDTA adsorbents, the percent of MB dye absorption increased with the amount of rC/AC3.7-EDTA. This may be due to the fact that higher dosages provide more sorption sites for the absorption of MB dye. On the other hand, when the rC/AC3.7-EDTA dose increased, the capacity of absorption of rC/AC3.7-EDTA adsorbents significantly decreased, reaching 0.914 from 34.21 mg/g, 15.678 from 43.92 mg/g, 23.014 from 45.26 mg/g, 28.64 from 57.05 mg/g, and 33.225 from 70.34 mg/g for 20, 40, 60, 80 and 100 mg/L MB dye concentration, respectively.

The presence of unsaturated sites on rC/AC3.7-EDTA adsorbents during MB dye adsorption is the effect of the decrease in MB dye adsorption capacity when the amount of rC/AC3.7-EDTA is raised from 0.05 to 0.25 g^54,55^. The *q*_e_ value decrease may be because of the splitting effect of flux between sorbets and sorbent with increasing rC/AC3.7-EDTA concentration leading to the reduction in the quantity of MB dye absorbed onto the unit weight of rC/AC3.7-EDTA produces a lower solute concentration is lower^41^.

#### Impact of starting MB dye concentration

**Figure 7:**
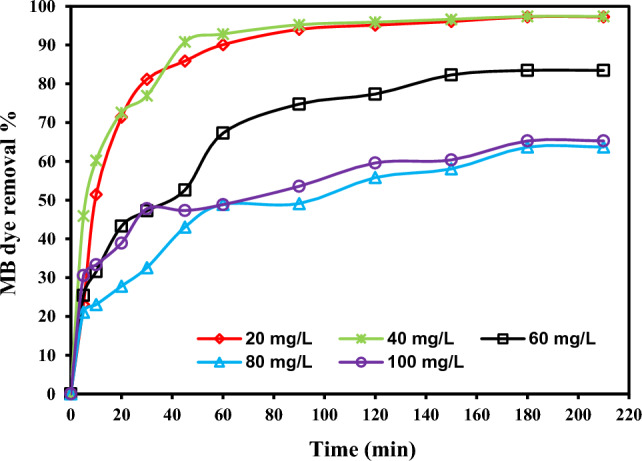
Impact of initial concentration on MB dye (*C*_0_: 20, 40, 60, 80 and 100 mg/L), adsorption using rC/AC3.7-EDTA adsorbent (2.0 g/L) (pH: 7, t: 210 min, T: 25 °C).

In addition to adsorbent dosage, it is thought that the starting MB dye concentration has a significant impact on the removal percentage and absorption capacity. The experiment was conducted at pH 7, adsorbent doses (0.2 g/100 mL), temperature 25 ± 2 °C, and at varying initial MB dye concentrations (20–100 mg/L) for 210 min. The results of the influent of starting concentration of MB dye are reported in Fig. 7, the plot of MB dye removal percentage with contact time at different starting concentrations of MB dye using a fixed adsorbent dosage of rC/AC3.7-EDTA (2.0 g/L). It is observed that with increasing starting MB dye concentration, the *q*_e_ increases from 9.91 to 32.63 mg/g for a rise in MB dye concentration from 20 to 100 mg/L using 2.0 g/L of rC/AC3.7-EDTA may be due to the strong driving force for mass transfer at high dye concentrations. On the other hand, the high ratio of active binding sites to the amount of MB dye molecules leading to appropriate absorbent-absorbate interaction may be responsible for the complete removal percentage of MB dye attained at such low MB dye concentrations. However, as the dye concentration was increased further from 20 to 100 mg/L, the removal percentage for rC/AC3.7-EDTA reduced from 99 to 65%, which was due to the active sorption sites being saturated as a result of the competing absorbate molecules on the surface of the absorbent^56^.

#### Impact of contact time

**Figure 8:**
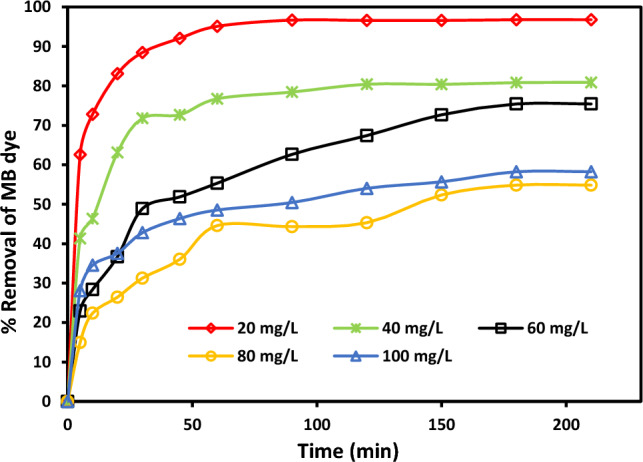
Contact time impact on MB dye absorption using rC/AC3.7-EDTA (C_0_: 20, 40, 60, 80 and 100 mg/L, m: 0.15 g, pH: 7, T: 25 °C).

The absorption of MB by rC/AC3.7-EDTA was studied at contact time over 240 min with different MB dye concentrations, and the results are illustrated in Fig. 8. The results revealed that as contact time increased, the amount of MB dye that was adsorbed increased as well. After 60 min, however, the removal rate was very low since so little MB dye was removed; the eliminated amount peaked at 180 min and remained steady after that. It was clearly found a high removal percent of MB dye reached 96.78% at pH 7 using 1.5 g/L of rC/AC3.7-EDTA of MB dye solutions with 20 mg/L MB dye initial concentration. The slow pore diffusion between the bulk of the absorbent and the solute was the reason for the slow rate of adsorption after 60 min^41^.

#### Impact of temperature

**Figure 9:**
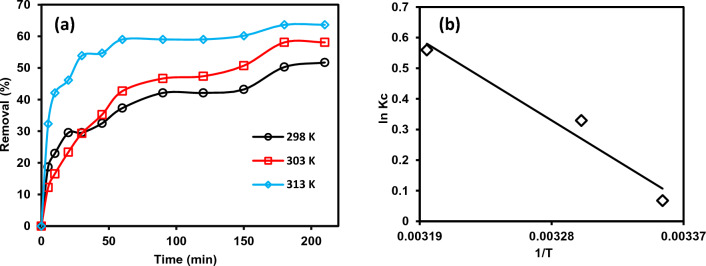
(**a**) Temperature impact on MB absorption using rC/AC3.7- EDTA adsorbents (m: 1.0 g/L, pH: 7, t: 210 min) and (**b**) Van’t-Hoff plot of ln(*Q*_e_/*C*_e_) against 1/T to determine thermodynamic parameters.

The impact of temperature on the absorption of MB dye is a Key factor as it controls the diffusion rate of dye molecules. The MB dye removal onto rC/AC3.7-EDTA adsorbents was studied at diverse temp. (298, 303 and 313 K) in order to investigate the thermodynamic parameters and absorption isotherms. Figure 9a presented the results indicating that the MB dye absorption capacity of rC/AC3.7-EDTA increased with increasing temperature from 298 to 313 K. When the temperature rises, the molecules of adsorbed MB dye engage more with the active sites on rC/AC3.7-EDTA, indicating that the absorption process is endothermic. Also, expanding the pore size due to high temperature may be another reason for enhancing the absorption capacities of MB dye^54,55^.

The values of *H*^0^ and *S*^0^ can be calculated from the Van’t-Hoff plot, as seen in Fig. 9b, and also from thermodynamic parameters in Table 1 for the MB dye absorption on the rC/AC3.7-EDTA. The resultant value *G*^0^ gives a negative value at different temperatures indicating that the MB dye absorption process onto the rC/AC3.7-EDTA was spontaneous. As well as, any increase in temperature leads to a more negative value of *G*^0^ as the absorption process is more promising at high temperatures. On the other hand, *H*^0^ (24,474.8 kJ/mol) gives a positive value indicating that the absorption was an endothermic process^54^. Additionally, *S*^0^ gives a positive value, indicating increased randomness at the interface between the solid and solution. Our results agree with Jabar et al.^55^, who mentioned that the chemically activated cocoa leaves biochar (CLs) showed adsorption to proceed towards the endothermic process. The increase in adsorption efficiency of CLs as temperature increased attested to the endothermic nature of the adsorption process. Moreover, a proportional decrease in Δ* G*^0^ with the continuous increase in temperature indicated the requirement of low internal energy by crystal violet dye to be adsorbed onto CLs as temperature increased^55^.

**Table 1:** Thermodynamic parameters for MB dye absorption onto rC/AC3.7-EDTA.

*T* (K)	∆*G*^0^ (KJ/mole)	∆H	∆S
298	− 49,213.8	24,474.8	83.017
303	− 49,628.9		
313	− 50,459.1		

### Regeneration

**Figure 10:**
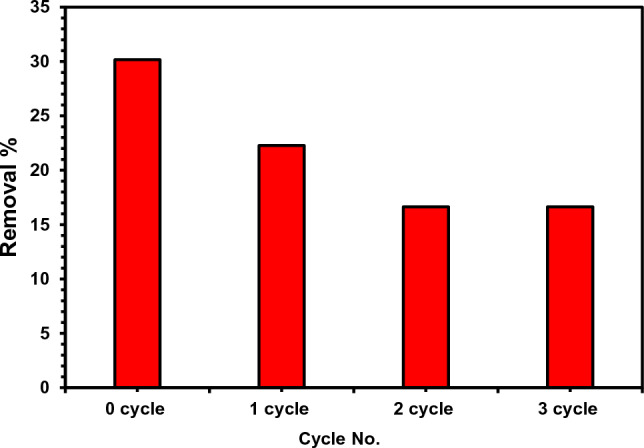
Adsorption capacity of methylene blue dye on rC/AC3.7-EDTA for three regeneration cycles.

The regeneration tests were assessed since the sorbent material's capacity to be reused is essential for lowering the net cost in practical applications. Recycling tests were carried out to investigate the RC/potential AC3.7-EDTA’s for reuse in the absorption of MB dye. By washing the dye that had been adhered to the nanofibers with ethanol and diluting HCl (0.1 M), the rC/AC3.7-EDTA nanofibers were renewed. The desorption-adsorption cycle was repeated 3 times. Figure 10 shows dye adsorption capacity for different cycles using 1.0 g/L of rC/AC3.7-EDTA. The adsorption capacity in cycle 1 was 21.41 mg/g, slightly lower than the 30.17 mg/g of the first use of rC/AC3.7-EDTA. Then the absorption capacity decreased to 16.57 mg/g after 3 cycles of regeneration.

### Modelling of adsorption isotherms

As indicated in Table 2, several mathematical models provide information on the physicochemical data for assessing the absorption process^57^. The result in Table 2 shows the applicability of the Langmuir isotherm model (LIM), which was the best-fitted isotherm model for removing MB dye onto rC/AC3.7-EDTA with high correlation coefficients *R*^2^ 0.991–1.00^41^. The maximum monolayer capacity (*Q*_m_) obtained from linear LIM is 60.61, 32.85, 30.58, 25.71 and 35.71 mg/g for 0.5, 1.0, 1.5, 2.0 and 2.5 g/L of rC/AC3.7-EDTA, respectively. The *Q*_m_ 60.61 mg/g of rC/AC3.7- EDTA is higher than those studied by^4^ 22.07 mg/g who studied the effect of Date Seed as a biosorbent, and near that 66.70 mg/g studied by^7^, who studied the impact of AC from peanut shells for acid yellow 36 removal.

**Table 2:** The coefficient isotherm parameters for MB dye sorption by rC/AC3.7-EDTA.

	Equation		Dose (g/L)	Parameter			References
Langmuir isotherm model	$\frac{{C_{e} }}{{q_{e} }} = \frac{1}{{K_{a} Q_{m} }} + \frac{1}{{Q_{m} }} \times C_{e}$ (6)			*Q*_*m*_	*K*_*a*_ × 10^3^	*R*^2^	^58,59^
			0.5	60.61	242.29	0.99	
			1.0	32.89	347.03	1.00	
			1.5	30.58	671.46	0.99	
			2.0	25.71	3382.61	1.00	
			2.5	35.71	666.67	0.99	
Freundlich isotherm model	${\text{Log}}q_{{\text{e}}} = \log K_{{\text{f}}} + 1/{\text{n}}\;\log C_{{\text{e}}}$ (7)			1/*n*	*K*_f_	*R*^2^	^60^
			0.5	0.12	4.32	0.99	
			1.0	0.18	3.19	0.93	
			1.5	0.22	3.11	0.94	
			2.0	0.22	2.77	0.97	
			2.5	0.2	3.24	0.97	
Temkin isotherm model	$q_{{\text{e}}} = \beta \ln A + \beta \ln C_{{\text{e}}}$ (8)			1/*n*_H_	*K*_H_	*R*^2^	^61^
			0.5	0.173	2.30*10^8^	0.97	
			1.0	0.241	5.06*10^4^	0.99	
			1.5	0.203	4.58*10^5^	1	
			2.0	0.143	8.47*10^8^	0.97	
			2.5	0.250	7.6*10^4^	0.99	
Halsey isotherm model	${\text{Ln}}\left( {q_{e} } \right) = \left[ {\left( \frac{1}{nH} \right)\ln K_{{\text{H}}} } \right] + \left( \frac{1}{nH} \right)\ln C_{{\text{e}}}$ (9)	Dose (g/L)	*A*_T_	*B*_T_	*b*_T_	*R*^2^	^62^
		0.5	956.17	4.32	573.9	1.000	
		1.0	12.73	4.97	498.92	0.990	
		1.5	1.08	10.20	242.97	1.000	
		2.0	4.20	18.12	136.73	0.990	
		2.5	21.41	5.62	441.19	0.990	
Generalized isotherm model	${\text{Ln}}\left( {\frac{Qm}{{qe}} - 1} \right) = \ln {\text{K}}_{{\text{G}}} - {\text{N}}_{{\text{G}}} \ln C_{{\text{e}}}$ (10)		*N*_b_	*K*_G_	*R*^2^		^63,64^
		0.5	0.21	149.42	0.970		
		1.0	0.27	890.64	0.990		
		1.5	0.29	727.78	1.000		
		2.0	0.24	690.88	1.000		
		2.5	0.27	533.09	0.990		

*C*_e_, Equilibrium concentration (mg/L); *q*_e_, The amount of oil sorbed (mg/g); *Q*_m_, Maximum sorption capacity (mg/g); *K*_a_, Sorption equilibrium constant (L/mg); *C*_e_, The concentrations of equilibrium dye during the solid and liquid phases (mg/L); *K*_F_ and 1/*n*, The Freundlich constants characteristics of the system. *T*, The absolute temperature in Kelvin; *R*, The universal gas constant; 8.314 J/mol K; *b*, Constant related to the heat of adsorption; *n*_H_ and *K*_H_, Halsey constants; *K*_G_, The saturation constant (mg/L); *N*_G_, The cooperative binding constant.

### Error functions investigation for best-fit isotherm model

Table 3 illustrates the error function of the isotherm models as the lowest accuracy is generalized isotherm model, and the best-fit isotherm models (IM) were Temkin and Halsey.

**Table 3:** Use various error functions to find the IM that best fits the experimental equilibrium results.

Error functions	Equation	Model	Value	References
Average percentage errors (APE)	${\text{APE}}\left( \% \right) = \frac{100}{N}\mathop \sum \limits_{i = 1}^{N} \left| {\frac{q\;e, isotherm - q\;e,calc}{{q\;e, isothem}}} \right|_{{\text{i}}}$ (11)	Langmuir	59.564	^65^
		Freundlich	15.533	
		Halsey	11.256	
		Generalizd	296.286	
		Temkin	10.770	
Hybrid fractional error (HYBRID)	${\text{HYBRID}} = \frac{100}{{N - P}}\mathop \sum \limits_{i = 1}^{N} \left| {\frac{q\;e, isotherm - q\;e,calc}{{q\;e, isothem}}} \right|_{{\text{i}}}$ (12)	Langmuir	60.622	^66,67^
		Freundlich	16.212	
		Halsey	11.256	
		Generalized	309.168	
		Temkin	12.224	
Chi-square error (X^2^ )	${\text{X}}^{2} = \mathop \sum \limits_{i = 1}^{N} \frac{{\left( {q\;e, isotherm - q\;e,calc} \right)^{2} }}{q\;e, isothem}$ (13)	Langmuir	11.655	^43^
		Freundlich	1.084	
		Halsey	0.583	
		Generalized	217.673	
		Temkin	0.516	
Sum of the squares of the errors (ERRSQ)	$$\begin{aligned} & {\text{ERRSQ}} = \mathop \sum \limits_{i = 1}^{N} \left( {{\text{q}}\;{\text{e}},\;{\text{calc}}{-}{\text{q}}\;{\text{e}},\;{\text{isotherm}}} \right)_{{\text{i}}}^{2}\end{aligned}$$ (14)	Langmuir	237.429	^65^
		Freundlich	35.352	
		Halsey	16.961	
		Generalized	6303.05	
		Temkin	13.348	
Marquardt’s percent standard deviation (MPSD)	${\text{MPSD}} = 100 \times \sqrt {\frac{1}{N - P}\mathop \sum \limits_{i = 1}^{N} \left( {\frac{q\;e, calc - q\;e,isotherm}{{q\;e,isotherm}}} \right)_{{\text{i}}}^{2} }$ (15)	Langmuir	98.201	^65^
		Freundlich	20.642	
		Halsey	21.372	
		Generalized	325.584	
		Temkin	16.579	
The sum of absolute errors (EABS)	${\text{EABS}} = \mathop \sum \limits_{i = 1}^{N} \left| {{\text{q}}\;{\text{e}},\;{\text{calc}}{-}{\text{q}}\;{\text{e}},\;{\text{isotherm}}} \right|_{{\text{i}}}$ (16)	Langmuir	11.926	^65^
		Freundlich	4.257	
		Halsey	2.579	
		Generalized	77.487	
		Temkin	2.659	
The root mean square errors (RMS)	${\text{RMS}} = 100 \times \sqrt {\frac{1}{N}\mathop \sum \limits_{i = 1}^{N} \left( {1 - \frac{q\;e, calc}{{q\;e,isotherm}}} \right)^{2} }$ (17)	Langmuir	96.133	^65^
		Freundlich	20.207	
		Halsey	21.372	
		Generalized	318.728	
		Temkin	16.579	

### Adsorption kinetic studies

**Figure 11:**
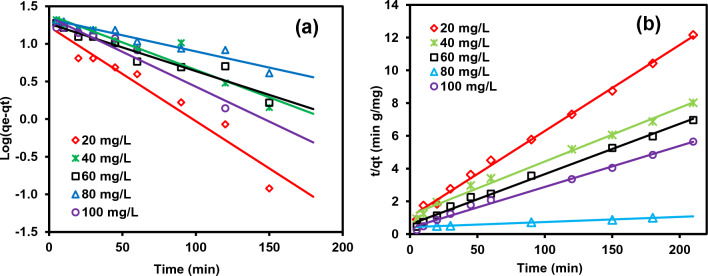
(**a**) Plot log(*q*_e_-*q*_t_) versus *t* of the PFOM and (**b**) Plot *t*/*q*t versus t of the PSOM for removal of MB dye (20–100 mg/L) by rC/AC3.7- EDTA (1.0 g/L).

Table 4 makes it abundantly evident that the data do not match the first-order kinetic model since all of the adsorption data’s estimated and experimental *q*_e_ values are extremely low, with poor *R*^2^ values (Fig. 11). On the other hand, the adsorption by rC/AC3.7-EDTA is more favorably by PSOM for MB dye with high calculated correlations equal to (1–0.991). The pseudo-second-order model with the highest *R*^2^ and the closest *q*_e_ (cal) to *q*_e_ (exp) (Table 4) best fits with the kinetic model according to Jabar and Odusote^68^. Similar observation was made by Jabar et al.^55^ in the kinetic study of the adsorption of crystal violet on CLs.

**Table 4:** The PFOM and PSOM absorption rate constants and calculated and experimental *q*_e_ values for various starting MB dye concentrations and 0.5 g/L of rC/AC3.7-EDTA.

Model	Equation	Parameter				References
Pseudo-first-order (PFOM)	$\log \left( {q_{{\text{e}}} - q_{{\text{t}}} } \right) = \log \left( {q_{{\text{e}}} } \right){-}\frac{{K_{1} }}{2.303}t$ (18)	*q*_*e*_ (exp.)	*q*_*e*_ (calc.)	*k*_*1*_ × 10^3^	*R*^*2*^	^42,68^
		34.21	18.60	16.12	0.99	
		43.92	27.54	6.91	0.96	
		45.26	23.69	11.75	0.98	
		57.05	10.80	12.67	0.95	
		70.34	43.22	41.91	0.82	
Pseudo second-order (PSOM)	$\left( {\frac{{\text{t}}}{{{\text{q}}_{{\text{t}}} }}} \right) = { }\frac{1}{{{\text{K}}_{2} \;{\text{q}}_{{\text{e}}}^{2} }} + \frac{1}{{{\text{q}}_{{\text{e}}} }}{ }\left( {\text{t}} \right)$ (19)	*q*_*e*_ (exp.)	*q*_*e*_ (calc.)	*k*_*2*_ × 10^3^	*R*^*2*^	^43^
		34.21	36.36	1.8	1	
		43.92	36.50	1.73	0.99	
		45.26	46.30	1.35	0.99	
		57.05	57.67	4.02	1	
		70.34	73.53	1.95	1	

*q*_e_, Sorption capacities at equilibrium (mg/g); *q*_t_, Sorption capacities at time t (mg/g); *k*_1_, Rate constant (L min^−1^); *K*_2_, The rate constant of sorption (g mg^−1^ min^−1^).

## Conclusion

A new rC/AC3.7-EDTA nanofiber adsorbent mat was fabricated to absorb of methylene blue dye. The experimental study showed that rC/AC3.7-EDTA nanofiber adsorbent mat had a high efficiency for absorption of MB dye with easy and good reusability. The optimum conditions for batch absorption studies of MB dye on rC/3.7AC-EDTA were observed at pH 7, and adsorption does 2.0 g/L and starting concentration of 20 mg/L after 180 min as contact time. Based on the optimal adsorption findings, the maximum removal percentage reached 99.14%. Adsorption followed the PSOM mechanism, according to the absorption kinetic studies. According to the isotherm investigations, the Langmuir model represented the absorption system of MB dye onto rC/AC3.7-EDTA. However, from the results obtained from error functions, the lowest accuracy IM is the generalized isotherm. The best fit IMs are Temkin and Halsey. The thermodynamic analyses also revealed that the process was endothermic and spontaneous. The results of this study show that rC/AC3.7-EDTA has outstanding performance and may be used as an effective absorbent for the uptake of dyes like MB dye from aquatic environments.

## Data Availability

The datasets used in this investigation are accessible for review upon request from the corresponding author of the paper.
